# Supplementary material for: DMRforPairs: identifying Differentially Methylated Regions between unique samples using array based methylation profiles
Source: BMC Bioinformatics. 2014 May 15;15:141. doi: 10.1186/1471-2105-15-141 (PMC4046028; doi:10.1186/1471-2105-15-141)
Supplement: Additional file 2 — DMRforPairs output for the comparison of A431-MCF7 and NA17018-NA17105. Please start from the HTML files in each folder. Available via the BMC Bioinformatics website. [file 1471-2105-15-141-S2.zip › 1394847754114233_MOESM2_ESM/A431_MCF7/figures/34106.pdf]

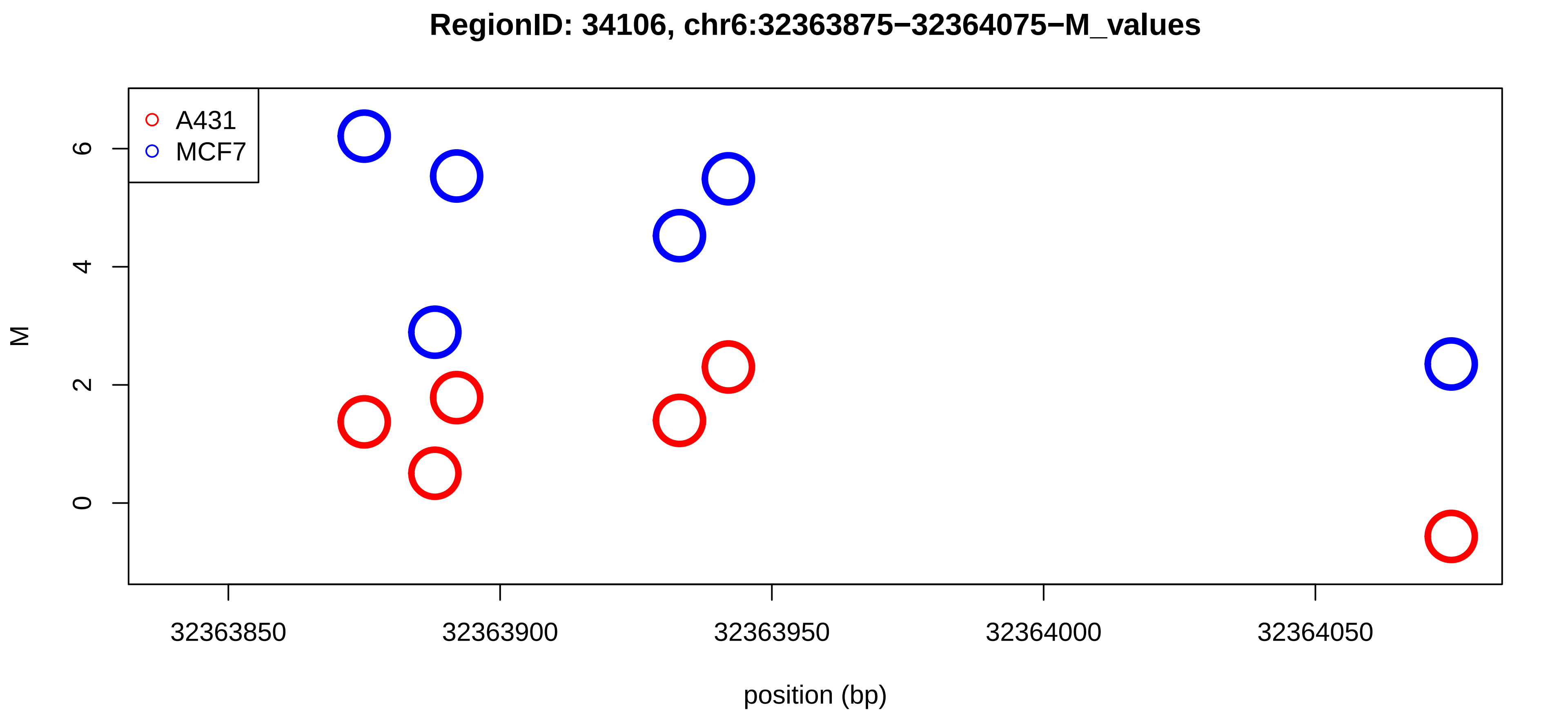
RegionID: 34106, chr6:32363875−32364075−M_values
A431
MCF7
M
0
2
4
6
32363850
32363900
32363950
32364000
32364050
position (bp)

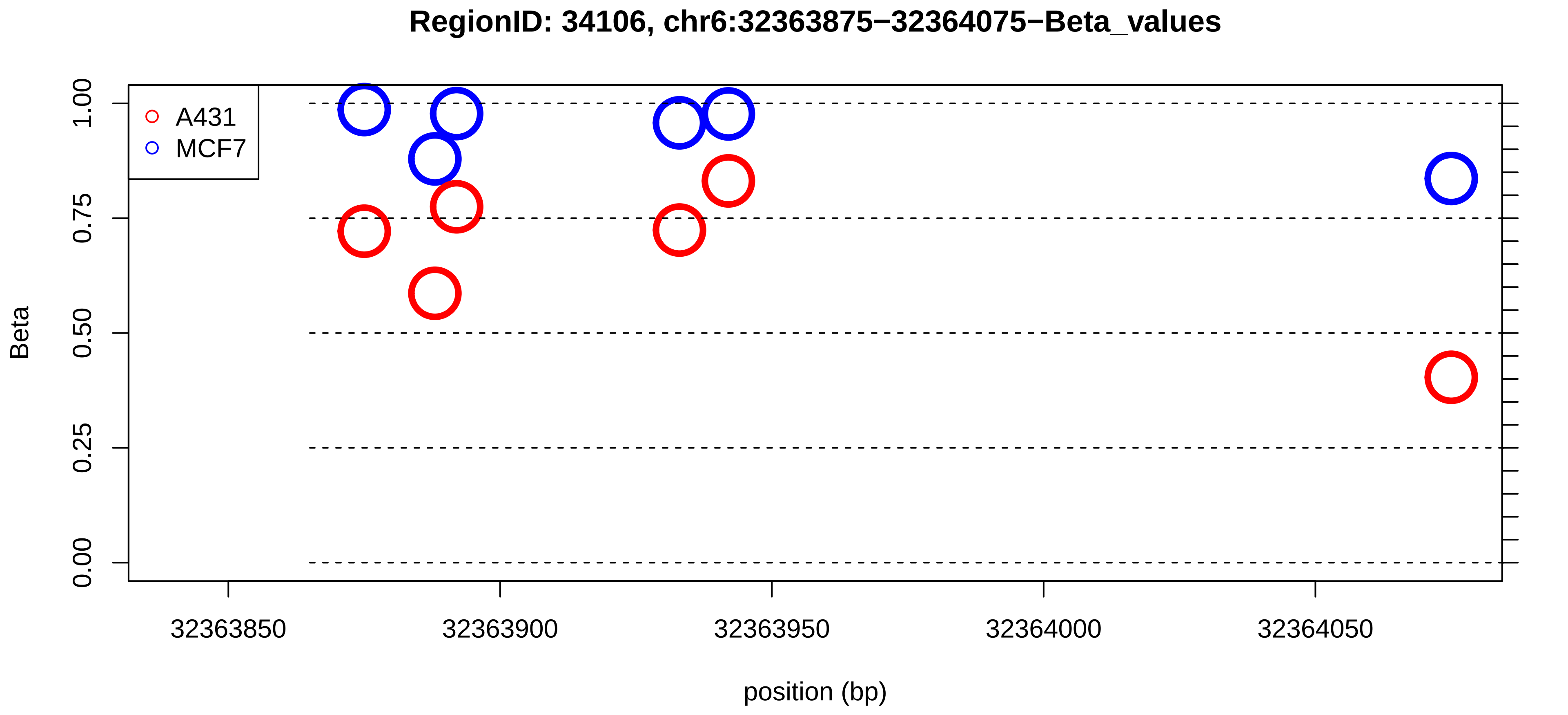
RegionID: 34106, chr6:32363875−32364075−Beta_values
A431
MCF7
Beta
0.00
0.25
0.50
0.75
1.00
32363850
32363900
32363950
32364000
32364050
position (bp)

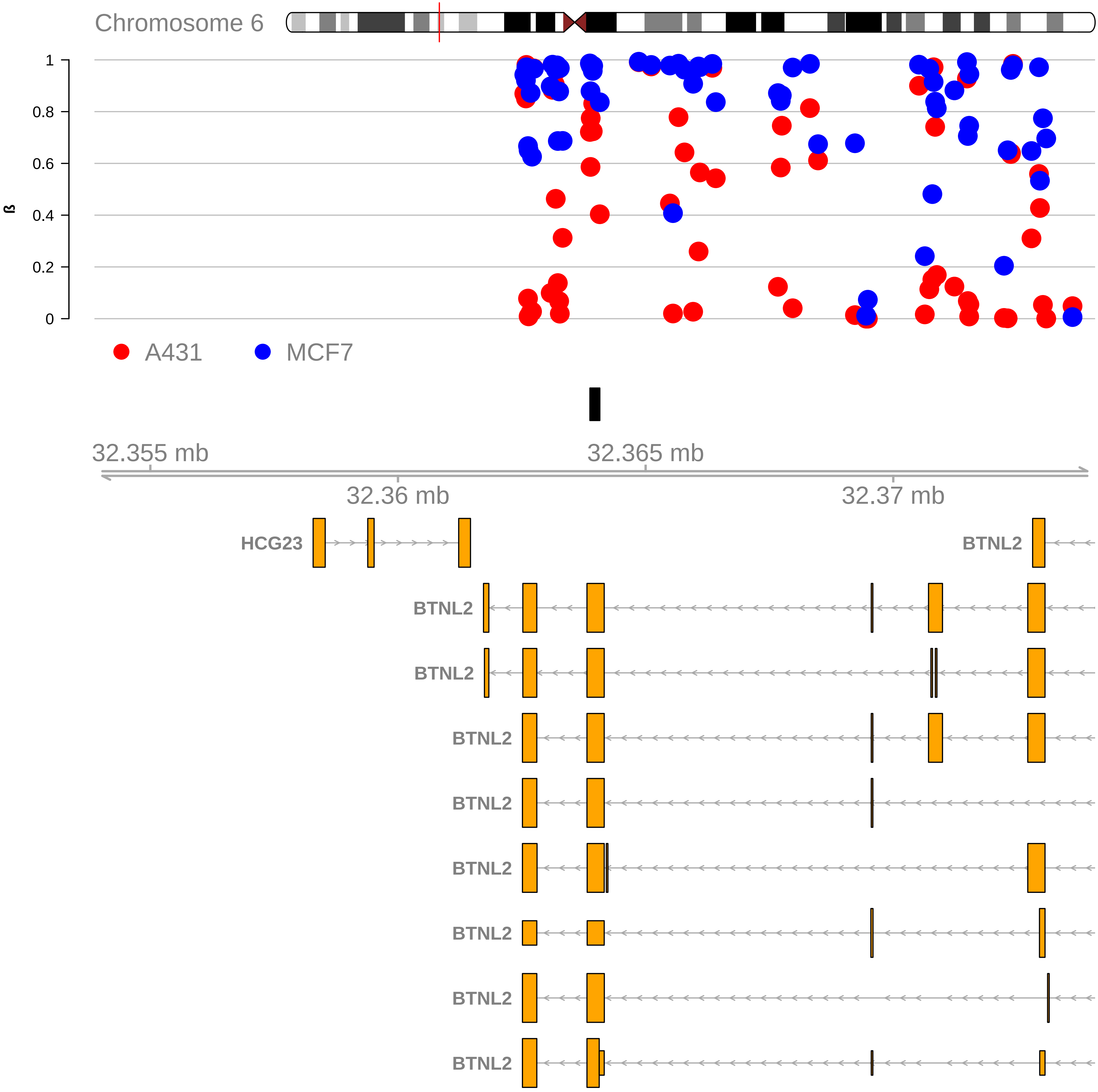

Chromosome 6
1
0.8
0.6
0.4
0.2
0
ß
A431
MCF7
32.355 mb
32.365 mb
32.36 mb
32.37 mb
HCG23
BTNL2
BTNL2
BTNL2
BTNL2
BTNL2
BTNL2
BTNL2
BTNL2
BTNL2
